# Precocious Locomotor Behavior Begins in the Egg: Development of Leg Muscle Patterns for Stepping in the Chick

**DOI:** 10.1371/journal.pone.0006111

**Published:** 2009-07-03

**Authors:** Young U. Ryu, Nina S. Bradley

**Affiliations:** Division of Biokinesiology and Physical Therapy, University of Southern California, Los Angeles, California, United States of America; Harvard University, United States of America

## Abstract

**Background:**

The chicken is capable of adaptive locomotor behavior within hours after hatching, yet little is known of the processes leading to this precocious skill. During the final week of incubation, chick embryos produce distinct repetitive limb movements that until recently had not been investigated. In this study we examined the leg muscle patterns at 3 time points as development of these spontaneous movements unfolds to determine if they exhibit attributes of locomotion reported in hatchlings. We also sought to determine whether the deeply flexed posture and movement constraint imposed by the shell wall modulate the muscle patterns.

**Methodology/Principal Findings:**

Synchronized electromyograms for leg muscles, force and video were recorded continuously from embryos while in their naturally flexed posture at embryonic day (E) 15, E18 and E20. We tested for effects of leg posture and constraint by removing shell wall anterior to the foot. Results indicated that by E18, burst onset time distinguished leg muscle synergists from antagonists across a 10-fold range in burst frequencies (1–10 Hz), and knee extensors from ankle extensors in patterns comparable to locomotion at hatching. However, burst durations did not scale with step cycle duration in any of the muscles recorded. Despite substantially larger leg movements after shell removal, the knee extensor was the only muscle to vary its activity, and extensor muscles often failed to participate. To further clarify if the repetitive movements are likely locomotor-related, we examined bilateral coordination of ankle muscles during repetitive movements at E20. In all cases ankle muscles exhibited a bias for left/right alternation.

**Conclusions/Significance:**

Collectively, the findings lead us to conclude that the repetitive leg movements in late stage embryos are locomotor-related and a fundamental link in the establishment of precocious locomotor skill. The potential importance of differences between embryonic and posthatching locomotion is discussed.

## Introduction

Chicks emerge from the egg after 21 days of incubation equipped to walk, swim and airstep [1]–[3]. Chicks begin moving embryonic day (E) 3. The movement (e.g., embryonic motility) is spontaneously generated throughout embryogenesis until hatching [4]–[6], which appears to be the only sensory-triggered behavior during embryonic development [5], [7], [8]. Embryonic motility is episodically generated by a recurrently connected excitatory network within the spinal cord that is transiently silenced by activity-dependent depression [9], [10]. Motility is initially driven by acetylcholine, then glutamate by E8-E10 [11]. The temporal features of the activity play an instructive role in motor neuron pathfinding [12], [13], and possibly the flexor-extensor and interlimb alternations for stepping [14]. However the relationship between the early network for motility and the locomotor network is uncertain [15], [16].

Leg movements during motility at E9 are characterized by alternating flexion and extension muscle synergies [17], [18] and joint excursions [19], [20]. Yet, electromyographic (EMG) and kinematic patterns appear to break down between E12 and E15 [17], [19], [21]. Recent studies indicate that a distinctly different pattern of repetitive leg movements (RLMs) emerges between E15 and E18 [22], [23]. RLMs are also composed of alternating flexion and extension, but the frequency range (1–10 Hz) far exceeds that for E9 motility (0.2–2 Hz). Interestingly, the RLM frequency range is similar to the combined ranges for three locomotor forms in hatchlings: walking, swimming and airstepping [2]. Together these findings raise the possibility that RLMs are an essential link between early motility and locomotion.

Leg muscles also express membership in either the flexor synergy for leg protraction (swing) or the extensor synergy for leg retraction (stance) during walking, swimming and airstepping [2]. Variations in muscle burst patterns across these behaviors appear to be due to differences in limb loading. For example, extensor burst duration exhibits a close association with step cycle duration during stance (walking), but a weak association during buoyancy (swimming) and limb suspension (airstepping). Differences in knee extensor activity also distinguish the three locomotor patterns from one another. During walking there are two knee extensor bursts; one in the latter part of swing to extend the knee and advance the foot, and one in late stance to propel the body forward [1]. Only the duration of the knee extensor burst in the stance phase co-varies with step cycle duration.

We reasoned that locomotor pattern generation must be established prior to hatching, given that chicks walk within hours afterwards. We also reasoned that RLMs might employ locomotor patterns given the similarities in cycle frequency range. However, in our previous study we observed that RLMs exhibited considerable variability in EMG activity [23]. This variability obscured ready identification of a fundamental pattern resembling any of the locomotor patterns observed in hatchlings. Thus, in this study we extend earlier findings by reporting quantitative analyses for the burst patterns at 3 ages. One aim was to determine if the EMG patterns for RLMs resemble any of the three locomotor patterns in hatchings. In addition, our earlier kinematic study of motility between E9 and E18 suggested that mechanical constraints due to growth of the body within a fixed egg volume increasingly alters joint kinematics between E15 and E18 [22]. Thus, differences we might find between locomotor and RLM muscle patterns could be attributable to constraints that include placement of flexor muscles at their shortest length and extensors at their greatest length. Therefore, another aim was to determine if EMG patterns would appear more similar to locomotor patterns when the shell constraint was removed. We provide evidence that EMG patterns for RLMs share some features common to locomotor behaviors at hatching, and the first evidence of alternating interlimb stepping in the embryo. We also provide evidence that the effects of shell removal were limited to the primary knee extensor, the only muscle that is known to distinctly vary its participation across the three locomotor behaviors in hatchlings [2].

## Results

We report EMG analyses for intralimb coordination based on 1206 RLM sequences. These data represent control conditions at E18 (386 RLM, 11 embryos) and E20 (353 RLM, 12 embryos), and the experimental condition, foot-free, at E20 (467 RLM, 9 embryos). The sample was drawn from a larger sample of 1569 RLMs whose rhythm properties were previously reported [23]. For these analyses, we excluded RLMs in which the tibialis anterior (TA), an ankle flexor and the reference muscle for our analyses, was the only rhythmically active muscle or if there were fewer than 10 rhythmically stable RLMs representing an experiment. EMG patterns at E15 are also reported (10 embryos).

### Leg muscle participation varied within and between experiments

**Figure 1:**
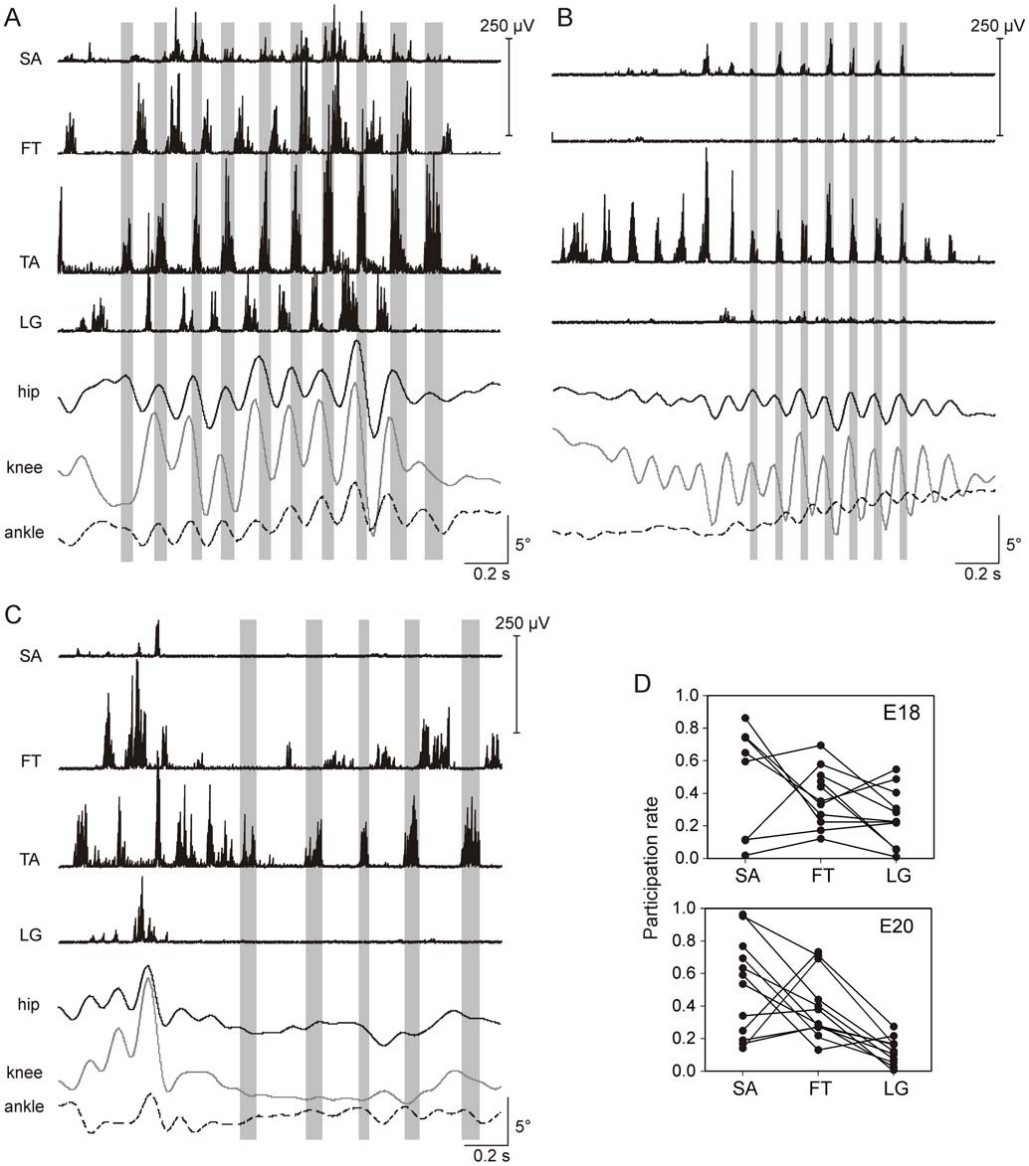
Variability in muscle participation during RLMs. RLMs from an E20 experiment are shown (A–C). *A:* This RLM consisted of 10 TA bursts and 9 rhythmically stable cycles (6.8±0.9 Hz). Vertical bars identify TA burst durations. Flexors (TA and SA) were coactive and reciprocally active with extensors (FT and LG). FT burst onsets preceded LG burst onsets. Flexors were active during flexion (downward deflections) of all 3 leg joints and extensors were active during extension (upward). *B:* Only TA and SA were active during 6 rhythmically stable RLM cycles (8.9±0.2 Hz). These cycles were preceded by several rhythmic TA bursts that did not meet criteria for either burst duration or rhythm stability (see Methods). Ankle excursions were minimal and opposite in direction of hip and knee. *C:* Only TA and FT were rhythmically active during 4 RLM cycles (4.1±0.7 Hz). Joint excursions were small and variable. *D:* The incidence of SA, FT, and LG bursts during RLMs is plotted for E18 (N = 11) and E20 (N = 12) embryos. The number of bursts detected by analysis methods was normalized to the total number of TA cycles per experiment (*participation rate*); 1 = always participated, 0 = never participated. Lines connect rates for each of the 3 muscles per experiment. SA was not implanted in 3 experiments (E18) and the LG implant was lost in 1 (E20). Abbreviations: SA, sartorius; TA, tibialis anterior; FT, femorotibialis; LG, lateral gastrocnemius.

The wide array of RLM rhythm frequencies and combinations of active muscles reported in our previous study raised the possibility that RLMs are a collection of rhythmic behaviors. Kinematic differences between RLMs, such as ankle motions that were in phase with proximal joints (Figure 1A) or out phase (Figures 1B, 3A) might be evidence of different limb behaviors. Quantitative analyses for this study extended those results revealing that the combination of active muscles varied markedly throughout every experiment, even in RLMs only seconds to minutes apart, though conditions were seemingly unchanged. In one E20 experiment for example, 4 leg muscles participated during one RLM (Figure 1A). Approximately 20 min later, a rhythmic sequence of TA bursts was spontaneously initiated that lacked rhythmic activity in the other EMG channels. It was followed immediately by a sequence of 6 TA cycles that included bursting in the sartorius (SA), a hip flexor (Figure 1B). In other RLMs during this experiment, TA sequences were accompanied by bursts in the femorotibialis (FT), a knee extensor (Figure 1C). To determine if there were any consistencies in recruitment, we determined the participation rate for each muscle across RLM cycles for each experiment. Based on within-subject averages (Figure 1D), the SA participated in nearly half of all RLM cycles at E18 (48%) and E20 (52%), FT participated in more than a third (38–40%), and the lateral gastrocnemius (LG), an ankle extensor, was least likely to participate at both E18 (26%) and E20 (13%). However, rates of participation for each muscle varied widely across experiments at both ages (Figure 1D).

**Figure 2:**
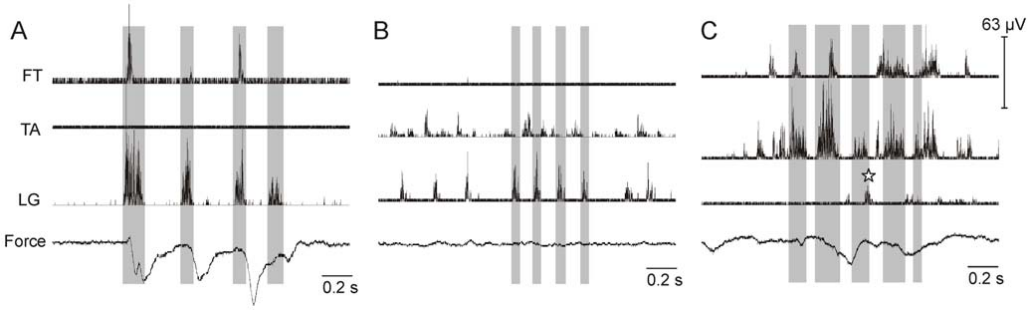
RLMs at E15. RLMs for 3 experiments are shown. A force transducer resting on the hip monitored general body displacements during recordings. Kinematic methods were not applied in these experiments. *A:* LG was rhythmically active for 3 cycles (3.2±1.0 Hz) and FT was coactive with LG. *B:* LG was rhythmically active for 3 cycles (6.4±0.5 Hz) and TA was reciprocally active with LG. *C:* TA was rhythmically active for 4 cycles (4.8±0.6 Hz), FT was coactive, and an LG burst was also detected by analysis methods (⋆).

RLM sequences were difficult to detect at E15 due to the low amplitude and irregular bursting that dominated recordings. Based on a stable burst rhythm in one muscle, though not necessarily TA, 160 EMG sequences (N = 10 embryos) were examined for muscle patterns. Participation of a second muscle was detected in 24 RLMs (15%). In these instances FT bursts occurred synchronously with repetitive LG bursting (Figure 2A); TA bursts alternated with LG (Figure 2B); or FT bursts occurred synchronously with TA (Figure 2C). Participation of a third muscle was observed only twice (Figure 2C; see also [23] Figure 1F). Thus at E15, rhythmic RLM bursting was more readily apparent than any RLM muscle pattern.

**Figure 3:**
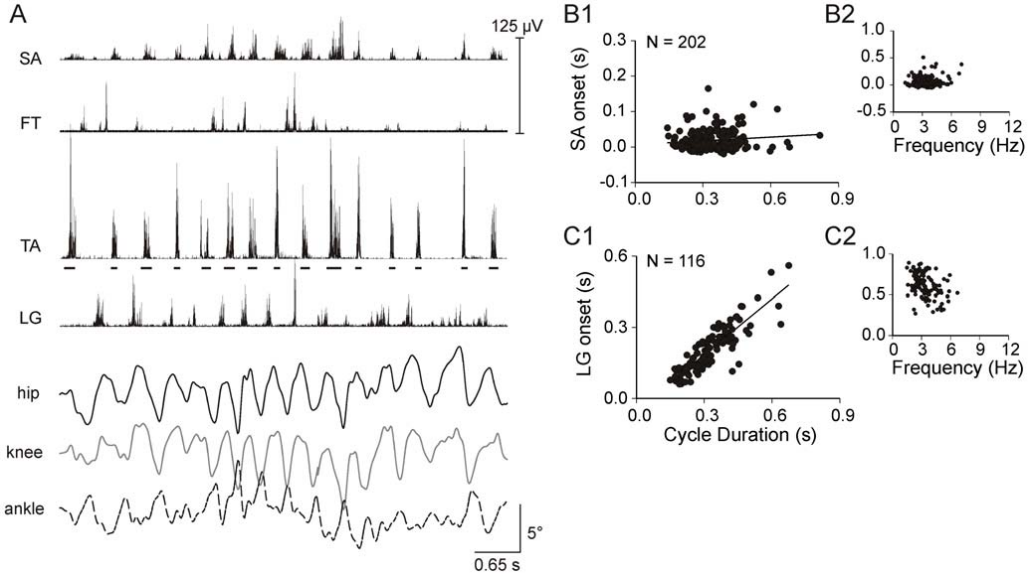
SA and LG bursts exhibited reliable onset patterns by E18. Data for an E18 experiment are shown. *A:* This RLM was 6.5 s long. Dashes underscore 15 TA bursts (2.4±0.5 Hz). TA amplitude was reduced 50% in this record to clearly visualize FT and SA traces. *B–C:* Larger scatter plots include trend lines for regression analyses. The number of bursts (N) in each trend analysis is indicated. In the small plots offset to the right, SA and LG burst onsets are normalized to the concurrent TA cycle duration (0–1) and plotted against TA cycle frequency (Hz). *B1:* SA burst onsets clustered near 0 s (slope = 0.03, R^2^ = 0.02). *B2:* SA relative onsets clustered early in the cycle (0–0.2) at all frequencies. *C1:* LG onset latency strongly co-varied with cycle duration (slope = 0.78, R^2^ = 0.76). *C2:* LG bursts clustered in the latter half of TA cycles with 82.8% of bursts falling between relative onsets of 0.5 and 1.0.

### RLM muscle patterns also exhibited reliable features

Though participation rates varied substantially across RLMs in an experiment, burst onsets for participating muscles were distinct. SA bursts began nearly synchronous with TA burst onset at both slower and faster RLM frequencies at both E18 and E20, with a mean onset latency of ±30 ms. An exemplary RLM and analyses for one E18 experiment are shown in Figure 3. The regression plot for SA burst onsets indicated SA onset latency clustered around 0 ms, the slope near 0.0 (Figure 3B1). Relative onsets fell mostly between 0–0.2 across a TA frequency range of 1–7 Hz (Figure 3B2). Slopes approximating 0.0 were found in 13 of 16 experiments at E18 and E20 combined. (See Table S1 in supplementary material for a detailed summary.)

When LG activity was well-formed, it alternated with TA bursts (Figure 3A). For example, regression results for the same E18 experiment indicated LG burst onsets co-varied closely with TA cycle duration (Figure 3C1). Relative onsets fell mostly in the latter half of the TA cycle at all RLM frequencies (Figure 3C2). These results were typical at both E18 and E20. LG onset closely varied with cycle duration in 6 of 9 experiments (R^2^>0.6), and was weak in only 2 (R^2^<0.4). Also, more than 60% of LG bursts began in the latter half of the TA cycle in 5 experiments (e.g., relative onset of 0.5–1.0), and no bias was found in 4 experiments. (See Tables S2 and S3 in supplementary materials for detailed summaries.)

**Figure 4 pone-0006111-g004:**
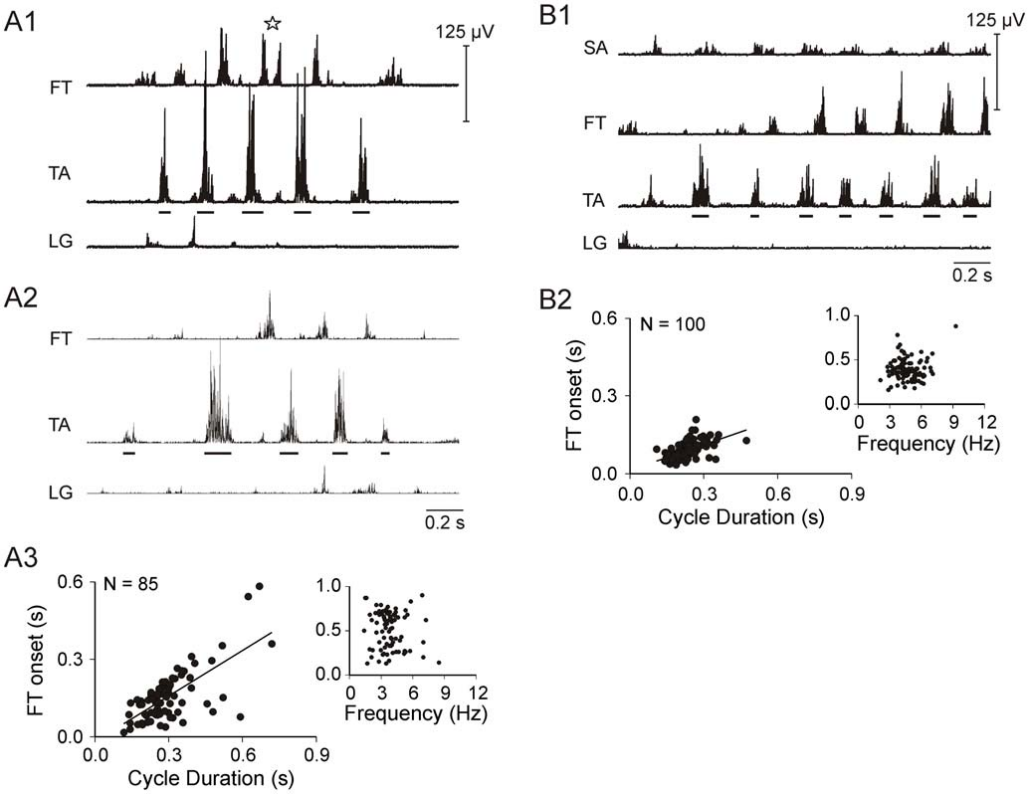
FT onset exhibited multiple trends. Data for an E18 (A) and E20 experiment (B) are shown. *A1:* FT bursts began in the first half of 4 TA cycles (3.9±0.5 Hz), accompanied by a 2^nd^ burst in cycle 3 (⋆). *A2:* In the next RLM 30 s later, FT bursts began in the latter half of TA cycles 2–4 (3.0±0.8 Hz). *A3:* FT onset and cycle duration (top) moderately co-varied (slope = 0.59, R^2^ = 0.48). Relative onset (bottom) was broadly distributed at all frequencies. 55% of FT bursts began in the latter half of the TA cycle. *B1:* FT bursts began in the first half of cycles 2–6 (4.2±0.6 Hz). *B2:* FT onset weakly varied with cycle duration (slope = 0.33, R^2^ = 0.33) and 84% of FT bursts began in the first half of the TA cycle.

The knee extensor, FT, also alternated with TA, but onset trends were distinct from LG. FT bursts exhibited 3 onset patterns relative to the TA cycle: early (relative onset <0.5), late (relative onset >0.5), or double bursting (early and late) at both E18 and E20. All 3 patterns were found within single experiments, as illustrated by 2 RLMs in Figure 4A. During the 1^st^ RLM (Figure 4A1) an FT burst began in the first half of each cycle, and included a double burst in cycle 3 (⋆). During the 2^nd^ RLM approximately 30 s later (Figure 4A2), FT bursts began in the latter half of cycles 2–4. FT burst onsets for this experiment modestly co-varied with TA cycle duration, and 2 clusters, early and late, were observed in the relative onset plot (Figure 4A3, bottom plot). FT burst onset varied closely with cycle duration in only 7 of 17 experiments (R^2^>0.6). FT onset varied weakly in 8 experiments (R^2^<0.4), as exemplified by the E20 experiment in Figure 4B. In the latter experiment, FT bursts began early in cycles 2–6 (Figure 4B1), and during the experiment relative onsets fell mostly between 0.2–0.5 over a frequency range of 2–8 Hz (Figure 4B2, bottom plot). More than 60% of FT bursts began in the first half of the TA cycle in 10 of 17 experiments, and a bias for the latter half of the cycle was found in only 4 experiments. FT findings did not differ between E18 and E20. (See Tables S2 and S3 in supplementary materials for detailed summaries.)

Burst durations were brief for all muscles at all TA burst frequencies. Average TA burst durations slightly exceeded 60 ms and averages for SA, LG, and FT ranged from 45 to 60 ms at E18 and E20. Regression analyses revealed that SA, LG, FT and TA burst durations did not vary with TA cycle duration. Both slope and R^2^ were approximately 0.0 in 89% of all burst duration analyses (N = 76 regressions, E18 and E20 combined).

### Foot-free

**Figure 5:**
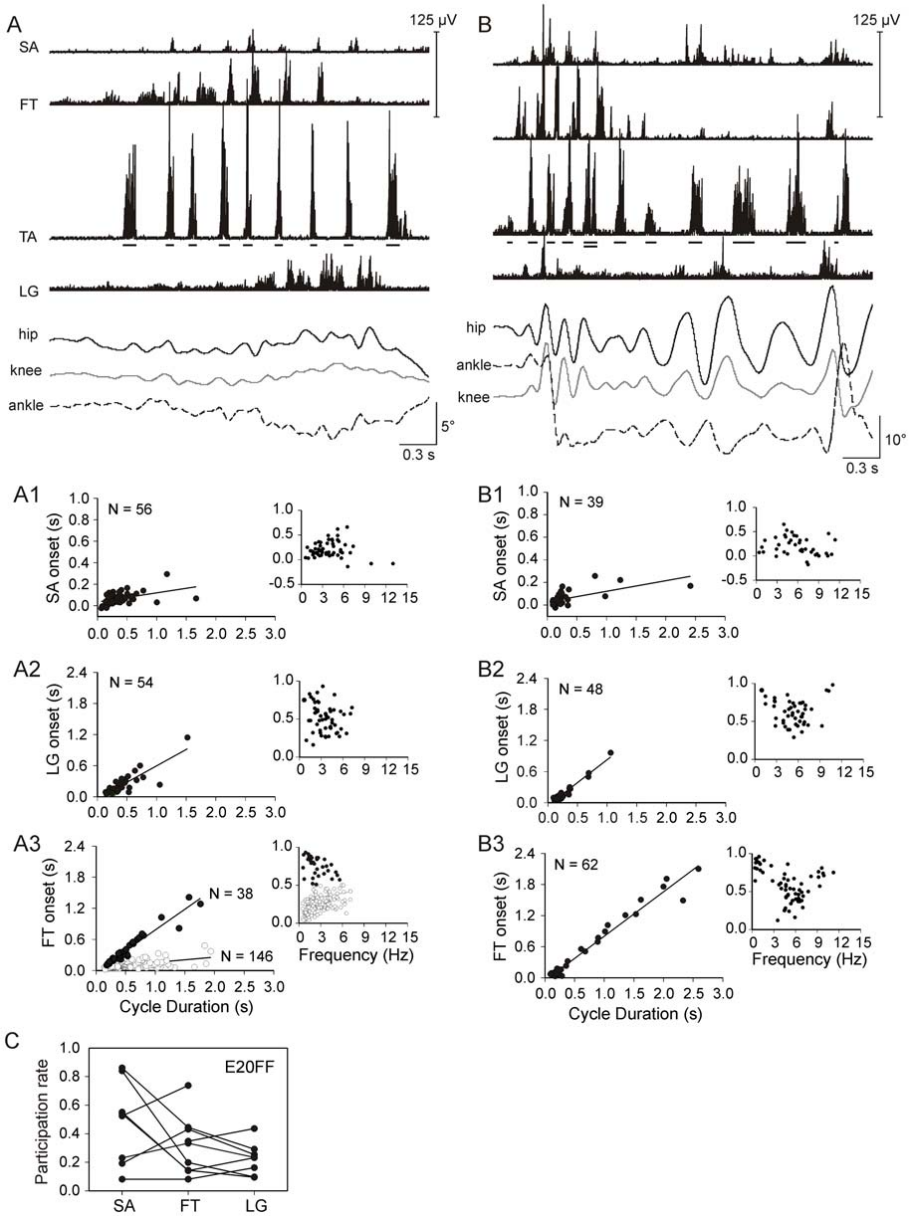
Control and foot-free RLMs during an E20 experiment. *A:* A control RLM of 8 cycles is shown (4.0±1 Hz). *B:* A double horizontal line identifies the end of one foot-free RLM (6.8±0.7 Hz) and start of another much slower RLM (3.1±0.7 Hz). Note differences in kinematic scales and also the faster frequencies during foot-free RLMs. *A1and B1:* SA onset latencies were similar for control (A1: slope = 0.08, R^2^ = 0.20) and foot-free RLMs (B1: slope = 0.09, R^2^ = 0.38). Relative onsets for SA were also similar. *A2 and B2:* LG onset strongly co-varied with cycle duration in both conditions (A2: slope = 0.63, R^2^ = 0.73; B2: slope = 0.89, R^2^ = 0.94). *A3 and B3:* Control FT burst onset (A3) displayed 2 regression trends (open circles: slope = 0.10, R^2^ = 0.30; closed circles: slope = 0.80, R^2^ = 0.93) that sorted by relative onset (open circles 0–0.5; closed circles 0.5–1.0). FT onset during foot-free RLM (B3) closely varied with cycle duration (slope = 0.86, R^2^ = 0.97), and exhibited a bias for late relative onset. *C:* SA, FT, and LG participation rates are shown for 9 foot-free experiments. The SA implant was lost in one experiment and the LG was in another.

Following removal of egg shell anterior to the foot (foot-free), E20 embryos extended the leg beyond the egg and produced larger joint excursion ranges during some RLMs (9 embryos). Compare the joint angle amplitudes for 2 RLMs from a single experiment during control (Figure 5A) and foot-free conditions (Figure 5B), noting differences in scale. There was also an increase in cycle frequencies greater than 6 Hz. However, few differences were observed in EMG patterns between conditions. For example, in the experiment shown, SA burst onset latencies mostly fell between 0–0.1 s, weakly varying with cycle duration during both conditions (Figures 5A1, 5B1). Similar SA results were obtained in 6 of 6 foot-free experiments. LG burst onset varied closely with cycle duration during control and foot-free RLMs (Fig. 5A2, 5B2). LG onset closely varied with cycle duration and a late relative onset was the predominant pattern in 4 of 6 foot-free experiments. Participation rates for foot-free experiments (Figure 5C) were also similar to E20 control data (Figure 1D).

FT onset was more likely to vary closely with TA cycle duration during foot-free experiments (5 of 7) than E20 control experiments (5 of 10). For example, FT burst onsets for the control data in the experiment shown in Figure 5 suggested there were 2 recruitment patterns (open and closed circles) across the entire range in cycle duration (Figure 5A3, left plot). The relative onset plot (Figure 5A3, right plot) revealed that the burst onsets sorted as early (open circles) and late (closed circles) in the TA cycle. The onset latency for early bursts was weakly associated with TA cycle duration; whereas, onset for late bursts was strongly associated. During foot-free RLM, early bursts were no longer observed at lowest RLM frequencies (Figure 5B3, right plot). Collectively, FT exhibited a distinct relative onset bias (>60% of sample) in 9 of 10 control experiments, and a preference for early onset (7 experiments). In contrast, only 4 of 7 foot-free experiments exhibited an FT relative onset bias, 2 early and 2 late (See Tables S2 and S3 for detailed summary comparisons). FT onset was also more likely to overlap TA activity during foot-free RLMs (33±11% of cycles) compared to control (15±15%, *p*<0.011), but burst durations (TA 65 ms, FT 55 ms) and duration of overlap (≈ 20 ms) were similar between conditions.

### Interlimb alternation during RLMs

**Figure 6:**
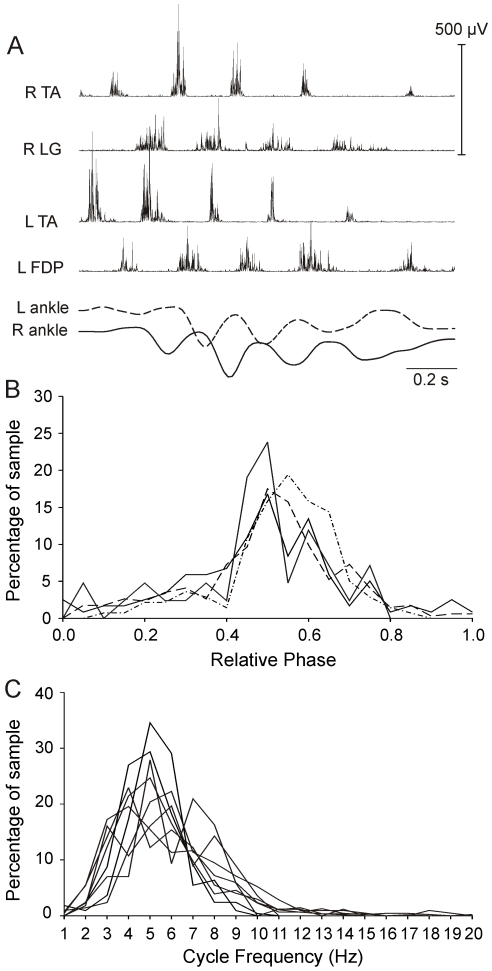
Interlimb coordinated during RLM at E20. Interlimb data are shown for 4 experiments at E20. *A:* A 3-burst sequence in the right (R) TA (3.4±1.2 Hz) alternated with a 4-burst sequence in the left (L) TA (4.0±0.7 Hz). R TA bursts also alternated with R LG bursts; L TA bursts alternated with left flexor digitorum profundus (L FDP) bursts. Anterior (upward)/posterior (downward) displacements of the 2 ankles were out of phase with one another. *B:* Left ankle extensor burst onsets were normalized to right extensor burst cycles (*relative phase*) and phase values were binned in 0.05 increments before plotting the distribution of interlimb phase patterns. The majority of left leg bursts fell between relative phases of 0.4–0.6 in all 4 experiments. *C:* The burst frequency distributions for the cycles plotted in B are shown for each leg (L TA and R TA). TA burst frequencies ranged from 1 to 10 Hz for both legs in each experiment.

Given some RLM features appeared to be similar to locomotor patterns while others were more ambiguous, we implanted leg muscles bilaterally at E20 in 4 experiments to determine if both legs were active during RLMs. We found that all experiments exhibited evidence of interlimb coordination. For example, in Figure 6A, the right TA and LG were alternately active, as were the right and left TA, and the right LG alternated with and the left flexor digitorum profundus (toe flexor/ankle extensor, FDP). The plot of ankle markers tracking anterior-posterior displacements indicated that the legs were moving in opposite directions, as during fore-aft foot trajectories while walking. Burst analyses for this experiment indicated that 56% of all cycles for the right LG (N = 211) were accompanied by bursts in the left LG or FDP. Results indicated that 50% of left FDP bursts began mid cycle (0.4–0.6). In the 4 experiments combined, EMG bursts were detected in the left leg during 49% to 62% of rhythmic sequences in the right leg, and the majority of left bursts began mid cycle (Figure 6B). A plot of burst frequencies for each leg (4 experiments) verified that these interlimb events fell within the RLM frequency range (Figure 6C).

## Discussion

### RLM patterns resemble locomotor patterns

Our first aim was to determine if EMG patterns for RLMs resemble any of the three locomotor patterns in hatchings. During walking, swimming and airstepping, several features are characteristic of leg muscle activity [1], [2]. Nearly all leg muscles exclusively participate in either the flexor synergy for limb protraction or extensor synergy for limb retraction. Burst onsets are close in time for flexor synergists (i.e., TA, SA). The ankle extensor (LG) alternates with flexors and the overlap between the bursts is little or none. In addition, activity of the knee extensor (FT) is distinctly different from other leg extensors. FT bursts twice per step cycle in walking and swimming, and once during airstepping; one FT burst begins during flexor activity in all 3 forms of locomotion. Our results indicated that EMG patterns for RLMs shared these features. SA burst onsets were closely associated with TA onset, regardless of TA burst frequency (Figure 3B). The ankle extensor, LG, was consistently recruited in the interval between TA bursts (Figures 1, 3). Recruitment of the knee extensor, FT, was distinct from LG; it most often began early in the cycle, but it could also burst late or twice per cycle (Figures 3–5). Also, like locomotion, RLMs could include alternating interlimb coordination (Figure 6). This array of similarities leads us to propose that RLMs in late-stage embryos are locomotor-related behavior.

Was there a match between the RLM burst pattern and any of the 3 locomotor forms at hatching? The RLM frequency range (1–10 Hz) embraced the combined cycle frequencies for the 3 locomotor behaviors at hatching [2]. On average, RLM frequencies at E18 (4 Hz) and E20 (5.4 Hz) were faster than the approximate averages for walking (2.8 Hz) and swimming (3.3 Hz), but airstepping (5.1 Hz) includes the higher frequencies common to RLMs. Though studies of locomotor patterns for hatchlings used the LG as reference for analyses, it also appears that the relative timing of burst onsets during RLMs closely resembled airstepping EMG [1], [2]. During RLMs, LG began in the latter half of the TA cycle and was immediately followed by the next TA burst (Figures 1, 3). FT began near TA offset and before onset of the next LG burst (Figures 1, 4–5). Extrapolating from published averages for airstepping (see [2] Figure 5), LG appears to begin in the later 40% of the TA cycle. A single FT burst begins near the end of the TA burst and terminates during the LG burst [2]. Further, during airstepping extensor burst durations are brief (<100 ms) and vary independent of cycle duration, a feature that was also typical of RLMs.

### Features that distinguish RLMs from locomotor behaviors

RLM EMG patterns also differed from airstepping. RLMs included burst frequencies below 3.5 Hz, a broader spectrum of FT onsets, and short lasting TA bursts that did not co-vary with cycle duration. Whereas during airstepping, TA burst durations are longer and scale with the step cycle [2]. The absence of covariation between extensor burst and cycle duration also distinguished RLMs from locomotion and swimming, for extensor bursts during stance (locomotion) and limb retraction (swimming) closely co-vary with the step cycle [2]. Thus, the absence of scaling between burst and cycle duration distinguished RLMs from all 3 posthatching forms of locomotion. The absence of burst scaling during RLMs could be an indication that proprioception at E18–E20 is not sufficiently mature to code movement dynamics [24]. However, longer extensor bursts are observed during hatching at E20 [17], possibly as embryos extend their legs into the shell wall to rotate their posture [25]. Thus, another possibility is that during most RLMs, the leg was not adequately loaded by foot contact with the shell to modulate extensor activity, for there were a few instances when LG burst durations were modestly lengthened (Figures 5A, 6A). On the other hand, flexor burst durations were probably not modulated by a load because flexor muscles operated in a shortened range due to the postural constraint imposed by the shell. It is also possible that descending pathways are required to modulate burst duration in ovo but that they do not modulate RLM activity.

### Shell removal did not enhance locomotor features of RLMs

We also considered the possibility that the extreme flexed posture imposed by shell constraint could mediate a set of cues that might mask locomotor patterns for airstepping, walking and/or swimming, and match force output to the limited movement space. The extreme flexed posture in ovo during the final days of incubation puts flexors at their most shortened length and all leg extensors at their greatest length. Thus our second aim was to determine if EMG patterns would appear more similar to one or more locomotor patterns when the shell constraint was removed. We reasoned that if leg movements were unconstrained, movements would be larger, plus flexor muscle lengths and loads would increase as extensor lengths decreased. However our results indicated that the temporal structure of RLM EMG activity was preserved, despite substantial increases in joint excursion range after shell removal (Figure 5B) and a significant increase in cycle frequency [23]. The resilience of the pattern during larger excursions was consistent with the observation that TA and SA burst onsets co-varied even when the ankle rotated out of phase with the hip and knee during control RLM (Figures 1B, 3A). FT onset was the only measure that exhibited a modest variation with shell removal. FT onset was more likely to co-vary with TA cycle duration, but relative onset was more ambiguous or shifted from an early to late burst in the TA cycle (Figure 5B3); and the incidence of briefly overlapping TA and FT activity significantly increased.

It was somewhat surprising to us that the EMG pattern for RLMs at E20 was not more mutable during the larger unconstrained motions, given the embryo would hatch and walk within 24 hrs. Any of the possible mechanisms discussed above might account for the minimal effect of shell removal. Also, the sensory cues for larger motions may have been insufficient because the embryo was in a side lying position and the leg flexors experienced less gravitational loading than if held vertically suspended, as during airstepping studies. Further, shell removal did not appreciably alter head/neck/spine flexion, and these inputs might act to reduce descending drive to spinal motor networks during foot-free as well as control experiments. On the other hand, it may be instructive that recruitment of FT, the muscle with the most varied pattern during control RLMs, was also the only muscle responsive to motion-related changes. The early and late onset variability in FT seems to be comparable to the onset shifts also seen in the vastus lateralis, a knee extensor in cat, during the ballistic motions of paw shaking [26], [27].

### RLM pattern and rhythm have different developmental time frames

In our previous study we propose that RLM rhythm generation may be modular because stable rhythms appeared to be isolated to individual muscles [23]. The same array and distribution of rhythms were observed by E15. Our present findings extend these results, suggesting that rhythm is established within a motor pool before it is expressed by an array of motor pools to form the RLM pattern. Participation of two muscles was infrequent at E15 but well established at E18. Studies of fictive locomotion and scratching in adult preparations have also observed that rhythmic activity in motor neuron pools can be sustained or resumed without temporal resetting as some motor pools drop out [28]–[31]. These observations led to proposals that central pattern generation for rhythmic limb movements involves two or more levels of control, i.e., one for rhythm and another for pattern (see [32] for a recent review). Thus, the differences in developmental time frame found for RLM rhythm and pattern generation in our studies appear to provide support for the proposal that pattern and rhythm generation involve multiple levels of control.

The developmental time frames for rhythm and pattern generation extend even more broadly than addressed by our present data. Repetitive limb movements are slow (0.2–2 Hz) at E9 [20], and temporally irregular between E12 and E15 [17], [19], [22]. The slow rhythms at E9 are likely produced by transient spinal network dynamics resulting from immature membrane properties [33], [34], and their progressive maturation may partially account for the temporal irregularities preceding emergence of RLM rhythms at E15. We do not know the mechanisms responsible for RLM rhythms, but descending pathways are well established by E15 [35], [36], and might provide excitatory drive as maturing spinal neurons lose their intrinsic excitability. Both slow and fast rhythms are also expressed in adult lamprey locomotion [37]. The slow frequencies are attributed to neural membrane properties, and the fast frequencies are attributed to recurrent excitation within the neural network (see [38] for a recent review). Our results suggest that the RLM pattern appears 1 to 3 days after RLM rhythm, however RLM pattern development may begin with the emergence of alternating flexor and extensor synergies by E9 [18]. Some of the subsequent transformations are likely attributable to maturation of membrane properties such as persistent inward currents [39]. However, the emergence of the FT double burst and its variants at E18 may require development of additional mechanisms such as another control layer in CPG circuitry [32]. Maturation of sensory inputs may also shape FT burst patterns [2]; however FT double bursting is retained in fictive locomotion after hatching, suggesting that it is centrally specified [40].

In sum, our present study of RLM burst patterns completes the first detailed studies of RLMs in the chick embryo. Findings revealed that despite considerable variations in the ensemble of active muscles during RLMs, burst patterns adhered to a common template. The template consisted of alternating flexion and extensor synergies, but also included burst patterns that distinguished knee extensor activity from ankle extensor activity. These findings are consistent with essential features of locomotor behaviors in hatchlings, and appear most similar to airstepping. Thus we conclude that RLMs in the late stage embryo are locomotor-related and produced by the developing neural circuits for locomotion. Our findings also suggest that RLM patterns are only modestly impacted by small modifications in physical constraint. Whether more dramatic changes in environmental conditions and postural context can significantly impact late stage embryonic behavior remains to be explored. Finally, our results lead us to conclude that RLM rhythm and pattern have different developmental time frames and that further study of their development may advance our understanding of locomotor control.

## Materials and Methods

Fertile Leghorn chicken eggs were incubated under standard conditions with a temperature-controlled humidified incubator (37.5°). Eggs were maintained under similar conditions in a temperature-controlled chamber during preparation and recording. The first day of incubation was E0 and the present studies were conducted at E15, E18 or E20. Age was verified at the end of experiments using staging criteria by Hamburger and Hamilton [41]. The viability of the embryo was assessed throughout the experiment by visually monitoring pulse rate and tracking body displacements with a force transducer (FT03C, Grass Instruments) placed in contact with the thigh [42]. If either indicator suggested the embryo was deteriorating, the experiment was terminated. All procedures were approved by the University Institutional Animal Care and Use Committee.

### EMG, video and force recordings

Embryos were prepared for synchronized electromyographic (EMG) and video recording of leg movements in the egg. A sagittal view of the leg was obtained by creating a window in the shell and dissecting egg membranes. Leg muscles were implanted with silver bipolar electrodes (o.d. 50 µm), including the sartorius (SA), a hip flexor; femorotibialis (FT), a knee extensor; tibialis anterior (TA), an ankle dorsiflexor; and lateral gastrocnemius (LG), an ankle extensor. The location of EMG electrode tips was later verified by dissection after euthanizing the embryo. In E18 and E20 embryos, modified minutin pins were inserted along the leg for digitizing hip, knee and ankle movements. In some experiments additional shell was removed anterior to the foot to reduce mechanical constraints on leg posture and movement (foot-free).

Synchronized EMG, force and video were recorded continuously for ≥3–4 hours (Datapac 2K2, Run Technologies) on a grounded anti-vibration table. EMG was band pass filtered (100–1,000 Hz), amplified (×2000), and digitally sampled (4 kHz). Force signals were low pass filtered (30 Hz), amplified (×20,000) and digitized (500 Hz).

### RLM Analyses

**Figure 7:**
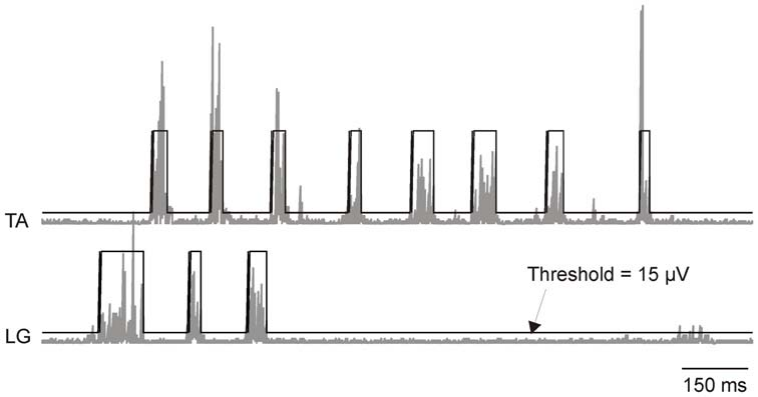
Burst detection methods. After signal rectification and baseline offset adjustment to 0 µV, average baseline channel noise was estimated during a quiescent interval of 100 ms. In the above example, a threshold of 15 µV (3 times baseline) for TA and LG burst detection is shown. EMG activity remaining above threshold for 20–400 ms and surrounded by subthreshold signal for 20 ms or longer was defined as a burst. Examples of burst detection are indicated by the upward square wave defections in the threshold trace.

Sequences of repetitive muscle bursting that were accompanied by leg movement were selected for analyses. The synchronized video and/or force recordings were used to confirm the presence of leg movements. The force recording was particularly useful for detecting RLM when joint motions were only a few degrees in magnitude due to spatial constraint but of sufficient force to produce rhythmic displacements of the body. EMG signals were rectified to detect burst onsets and offsets based on 3 criteria: burst threshold (2–4 times baseline amplitude), burst duration (20–400 ms), and inter-burst interrupt (20 ms) (Datapac 2K2). Baseline amplitude for each EMG channel was estimated from a quiescent 100 ms segment of recording at the start of the experiment. The threshold for each EMG channel was determined by pilot analyses of several RLMs and based upon the combined parameter set that captured the greatest number of EMG bursts. An example of the burst detection method is shown in Figure 7.

EMG burst and cycle measures were referenced to TA burst onset, because TA was the most reliably rhythmic of the muscles recorded [23]. Thus, *TA cycle duration* was defined as the time between consecutive TA burst onsets. *Burst onset latencies* for all other muscles were measured from onset of the preceding TA burst, with the exception of SA bursts that slightly preceded the concurrent TA burst. Latencies were divided by the concurrent TA cycle duration to obtain *relative onset latencies*. The time between offset of TA bursts and onset of antagonist muscles (FT, LG) was also calculated to determine if alternating bursts exhibited some duration of *co-activity* with TA. Leg movement during the RLM was digitized and joint angles were calculated for display with the EMG traces using methods established for in ovo recordings [22], [43].

Analyses were limited to RLM cycles that were rhythmically stable (SD≤1 Hz) for 4 or more consecutive TA bursts [23]. Regression analyses were used to examine linear trends in EMG burst parameters relative to TA cycle duration within an experiment, and coefficients of determination (R^2^) are reported. Data sets containing fewer than 20 bursts were excluded. The t-test for independent samples was used to compare subject means between age groups (E18 and E20) and conditions (control and foot-free). A *p*<0.05 was considered significant.

## Supporting Information

Table S1 Slope results for regression analyses of SA onset time vs. cycle duration.
(0.03 MB DOC)

Table S2 R^2^ regression coefficients for extensor burst onset time vs. cycle duration.
(0.03 MB DOC)

Table S3 Relative onset trends for LG and FT.
(0.03 MB DOC)
