# Supplementary material for: Precocious Locomotor Behavior Begins in the Egg: Development of Leg Muscle Patterns for Stepping in the Chick
Source: PLoS One. 2009 Jul 3;4(7):e6111. doi: 10.1371/journal.pone.0006111 (PMC2700958; doi:10.1371/journal.pone.0006111)
Supplement: Table S1 — Slope results for regression analyses of SA onset time vs. cycle duration. (0.03 MB DOC) [file pone.0006111.s001.doc]

**Table S1.** Slope results for regression analyses of SA onset time vs. cycle duration.

| slope | E18 (N=5)^1^ | E20 (N=11)^1^ | E20FF (N=6)^1^ |
| --- | --- | --- | --- |
| < 0.1 | 4 | 9 | 4 |
| ≥ 0.1, <0.3 | 1 | 2 | 2 |

N=number of experiments with sufficient sample for regression analyses.
