# Supplementary material for: Precocious Locomotor Behavior Begins in the Egg: Development of Leg Muscle Patterns for Stepping in the Chick
Source: PLoS One. 2009 Jul 3;4(7):e6111. doi: 10.1371/journal.pone.0006111 (PMC2700958; doi:10.1371/journal.pone.0006111)
Supplement: Table S2 — R2 regression coefficients for extensor burst onset time vs. cycle duration. (0.03 MB DOC) [file pone.0006111.s002.doc]

**Table S2.** R^2^ regression coefficients for extensor burst onset time vs. cycle duration.

|  | LG |  |  |  | FT |  |  |
| --- | --- | --- | --- | --- | --- | --- | --- |
| R^2^ | E18 (N=6) | E20 (N=3) | E20FF (N=6) |  | E18 (N=7) | E20 (N=10) | E20FF (N=7) |
| ≥ 0.6 | 4 | 2 | 4 |  | 2 | 5 | 5 |
| ≤ 0.4 | 1 | 0 | 1 |  | 4 | 4 | 1 |
| >0.4, <0.6 | 1 | 1 | 1 |  | 1 | 1 | 1 |
