# Supplementary material for: Precocious Locomotor Behavior Begins in the Egg: Development of Leg Muscle Patterns for Stepping in the Chick
Source: PLoS One. 2009 Jul 3;4(7):e6111. doi: 10.1371/journal.pone.0006111 (PMC2700958; doi:10.1371/journal.pone.0006111)
Supplement: Table S3 — Relative onset trends for LG and FT. (0.03 MB DOC) [file pone.0006111.s003.doc]

**Table S3.** Relative onset trends for LG and FT.

|  | LG |  |  |  | FT |  |  |
| --- | --- | --- | --- | --- | --- | --- | --- |
| onset bias | E18 (N=6) | E20 (N=3) | E20FF (N=6) |  | E18 (N=7) | E20 (N=10) | E20FF (N=7) |
| Early^1^ | 0 | 0 | 0 |  | 3 | 7 | 2 |
| Late^2^ | 4 | 1 | 4 |  | 1 | 2 | 2 |
| Equal^3^ | 2 | 2 | 2 |  | 3 | 1 | 3 |

1. More than 60% of bursts began in the first half of the TA cycle. 2. More than 60% of bursts began in the latter half of the TA cycle. 3. Relative onsets were equally distributed.
